# Design of Novel Fe-Doped NiCo-LDH/NiFeCo-Oxide Composite Nanosheets Grown on Carbon Fiber Cloth for High-Performance Flexible Asymmetric Supercapacitor

**DOI:** 10.3390/ma19091747

**Published:** 2026-04-24

**Authors:** Wenyi Qiu, Zuo Zhu, Xiaoming Li, Hongwei Luo, Junfeng Chen, Chen Wang, Linchi Zou

**Affiliations:** 1 School of Materials Science and Engineering, Fuzhou University, Fuzhou 350108, China; 2 Laboratory of Green and Efficient Development of Phosphorus Resources, Fuzhou University, Fuzhou 350108, China; 3 School of Materials Science and Engineering, Fujian University of Technology, Fuzhou 350118, China

**Keywords:** NiCo-LDH, Fe doped, hierarchical structure, flexible electrode, supercapacitors

## Abstract

**Highlights:**

- Designed a novel Fe-doped NiCo-LDH/NiFeCo-oxide composite for flexible supercapacitors.
- NiFeCo-LDH/NiFeCoO@CC shows quadruple NiCo-LDH capacity with stable cycling.
- NiFeCo-LDH boosts reaction, while NiFeCoO@CC improves conductivity and stability.
- The heterointerface between LDH and metal oxides further boosts conductivity.

**Abstract:**

Layered double hydroxides (LDH) demonstrate significant potential in flexible superca-pacitors due to their high energy storage capability and adjustable architectures. Never-theless, the practical specific capacitance exhibited by current LDH remains below expec-tations, which is attributed to suboptimal electrode performance and limited active sites. Herein, a novel Fe-doped NiFeCo-LDH/NiFeCoO nanosheet composite supported on car-bon cloth was designed and fabricated as a flexible electrode. In this composite, the Ni-FeCo-LDH supplies numerous reactive centers and accelerates electrochemical kinetics, while the NiFeCoO and carbon cloth significantly improve electrical conductivity and cy-cling stability. Moreover, the heterointerface formed between the LDH and the metal oxide phase further facilitates charge transfer. Owing to such synergistic interactions, the pre-pared NiFeCo-LDH/NiFeCoO@CC electrode demonstrates an excellent areal specific ca-pacitance of 3.282 F cm^−2^ at a current density of 1 mA cm^−2^, while maintaining a high ca-pacity preservation reaching 88.09% following 5000 cycles. Furthermore, the assembled NiFeCo-LDH/NiFeCoO@CC//AC asymmetric supercapacitor delivers an outstanding en-ergy density reaching 0.302 mWh cm^−2^ under a power density of 0.776 mW cm^−2^, coupled with an excellent capacitance preservation of 85.29% over 5000 cycles. Meanwhile, it can maintain its initial capacitance under varying bending degrees, rendering it widely ap-plicable for future advanced flexible and wearable electronic devices.

## 1. Introduction

The accelerated depletion of fossil fuel reserves has induced energy shortages that severely impede the sustainable advancement of global industrialization. Consequently, the development of high-performance energy storage technologies has emerged as an urgent imperative [1,2,3,4]. Within the diverse range of energy storage systems, flexible solid-state supercapacitors are attracting substantial interest for portable power devices, driven by their seamless integration of physical bendability along with superior electrochemical performance [5,6,7,8].

The design and preparation of active components are generally regarded as crucial factors affecting the energy storage capabilities of ultracapacitors [9]. Therefore, significant efforts have focused on developing high-performance electrode materials, including polymers, carbon-based frameworks, and transition-metal sulfides/oxide derivatives [10]. Among these electrode materials, Layered double hydroxides (LDHs) have garnered immense interest because of their exceptional redox activity, tunable chemical composition, and unique layered structure. In particular, NiCo-LDH stands out as an appealing active candidate because of its superior calculated charge-storage capability [11,12]. However, the practical specific capacitance of NiCo-LDH frequently falls below theoretical expectations, which is mainly attributed to its relatively poor electronic conductivity and a tendency to agglomerate during synthesis.

Doping NiCo-LDH with third metal elements such as Fe, Mn, Zn and Cr to form ternary LDH can effectively mitigate the insufficient exposure of active sites arising from agglomeration through multi-metal synergistic effects [13]. Fe possesses different valence states, which can enhance the redox reactivity of the material, thereby delivering superior capacitive performance [14]. However, sole Fe doping still fails to fundamentally address the performance degradation of NiCo-LDH caused by structural collapse during cycling. To further enhance the structural stability of electrode materials, the construction of heterostructures has been demonstrated as an effective and feasible strategy [15,16]. Bai et al. [17] reported that metal oxides exhibit relatively low electronegativity, and the cations within their structure can bond with cations in LDH. Therefore, the integration of Fe-doped NiCo-LDH with metal oxides is theoretically expected to effectively enhance both capacitive performance and cycling stability [18].

In this work, a novel heterostructure composite comprising Fe-doped NiCo-LDH and NiFeCoO was designed and fabricated. The crystallinity, composition, morphology and interfacial structure of the resulting NiFeCo-LDH/NiFeCoO@CC composites were systematically characterized, and the synergistic effects among components on electrochemical performance were investigated. This work provides an innovative strategy for developing advanced electrode materials with promising prospects in supercapacitor applications.

## 2. Experiments

### 2.1. Fabrication of NiFeCoO@CC

Firstly, a mixed solution containing Ni(NO_3_)_2_·6H_2_O (6 mM, Xilong Scientific Co., Ltd., Shantou, China), Fe(NO_3_)_3_·9H_2_O (2 mM, Sinopharm Chemical Reagent Co., Ltd., Shanghai, China), Co(NO_3_)_2_·6H_2_O (4 mM, Sinopharm Chemical Reagent Co., Ltd.), NH_4_F (20 mM, Xilong Scientific Co., Ltd.) and urea (40 mM, Xilong Scientific Co., Ltd.) was prepared under continuous magnetic stirring for 2 h. Following this, the pre-treated carbon cloth (CC, Qian Ding Li Electronic Technology Co., Ltd., Shenzhen, China) was transferred into an autoclave filled with 40 mL of the above mixture, then heated to 160 °C and maintained for 8 h to facilitate hydrothermal growth. After cooling to room temperature, the obtained product was rinsed repeatedly with deionized water and ethanol. The resulting sample was denoted as NiFeCo-LDH@CC.

Subsequently, the as-obtained NiFeCo-LDH@CC was dried under vacuum at 6 °C for 4 h. The intermediate product was then calcined in air at 350 °C for 2 h. After the heat treatment, the product was collected and washed thoroughly with deionized water and ethanol several times. Finally, it was dried under vacuum at 60 °C for another 12 h, and the resulting sample was denoted as NiFeCoO@CC.

### 2.2. Fabrication of NiFeCo-LDH/NiFeCoO@CC

**Figure 1:**
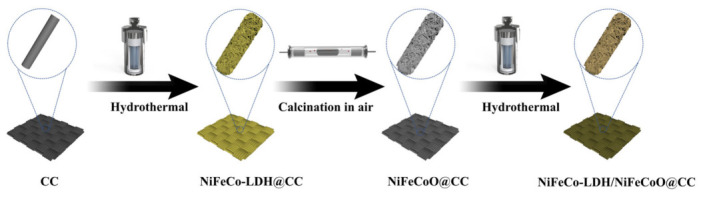
Schematic illustration of NiFeCo-LDH/NiFeCoO@CC composites.

A mixed solution containing Ni(NO_3_)_2_·6H_2_O (6 mM), Fe(NO_3_)_3_·9H_2_O (2 mM), Co(NO_3_)_2_·6H_2_O (4 mM), NH_4_F (20 mM) and urea (40 mM) was magnetically stirred for 120 min. Subsequently, NiFeCoO@CC was immersed in 40 mL of this precursor solution within an autoclave and subjected to hydrothermal treatment at 160 °C for 8 h. After cooling to room temperature, the specimens were rinsed repeatedly with deionized water and ethanol and vacuum-dried at 60 °C for 12 h. The resulting composite was designated NiFeCo-LDH/NiFeCoO@CC. The synthetic procedure is illustrated in Figure 1. Additionally, the electrode without Fe(NO_3_)_3_·9H_2_O was also fabricated under identical conditions for comparison. The obtained sample was designated as NiCo-LDH/NiFeCoO@CC.

### 2.3. Materials Characterization

X-ray diffraction (XRD, Ultima III, Rigaku, Tokyo, Japan) was employed to obtain crystallographic information of the as-synthesized products. Field-emission scanning electron microscopy (SEM, Zeiss Supra 55, Oberkochen, Germany) combined with transmission electron microscopy (TEM, FEI-Talos F200X, Hillsboro, OR, USA) was utilized to acquire surface morphology images and elemental mapping data. X-ray photoelectron spectroscopy (XPS, ESCALAB 250Xi, Thermo Fisher, Waltham, MA, USA) was applied to analyze the oxidation states and elemental composition of the prepared samples. A FT-IR spectrometer (Nicolet 5700, Thermo Fisher, Waltham, USA) was used to record the infrared spectra, from which the chemical bonds and functional groups were deduced. Textural parameters, including the BET surface area and pore structure, were acquired with a surface area and porosity analyzer (ASAP 2460, Micromeritics, Norcross, GA, USA).

### 2.4. Electrochemical Measurements

All electrochemical evaluations were performed utilizing a conventional three-electrode setup. The working electrode was the synthesized specimen, where a Hg/HgO electrode and a platinum plate functioned as the reference and auxiliary electrodes, respectively. The GAMRY Interface 1000E workstation (Gamry Instruments, Warminster, PA, USA) was used to monitor the cycling durability, while the PARSTAT MC unit (Princeton Applied Research, Oak Ridge, TN, USA) was employed for cyclic voltammetry (CV), galvanostatic charge–discharge (GCD), and electrochemical impedance spectroscopy (EIS) measurements. An aqueous 2 M KOH solution was utilized as the testing electrolyte throughout.

The CV measurements were run from 0 to 0.6 V with sweep speeds of 5–50 mV s^−1^. GCD experiments were carried out at 1, 2, 5, 7, and 10 A g^−1^ as well as at 1, 2, 5, 7, and 10 mA cm^−2^. The various electrodes delivered a maximum working voltage spanning from 0.465 to 0.51 V. EIS data were collected from 10^−1^ to 10^4^ Hz with a 10 mV AC signal. Long-term cycling tests consisted of 5000 consecutive charging and discharging processes at a current density of 10 mA cm^−2^. Equation (1) was used to calculate the area-normalized capacitance (C_a_, F cm^−2^):

(1) $$C_{a}=\frac{I\times t}{s\times ∆V}$$

where I indicates the response current (A), t refers to the discharging time (s), S denotes the geometric electrode area (1 cm^2^), and ΔV stands for the operating voltage window (V).

The asymmetric supercapacitor (ASC) device was constructed using NiCo-LDH/NiFeCoO@CC and activated carbon (AC). Following the principle of charge balance, the specific weight loadings for the positive electrode and anode within the ASC are determined by Equation (2):

(2) $$\frac{m_{+}}{m_{-}}=\frac{C_{-}\times ∆V_{-}}{C_{+}\times ∆V_{+}}$$

where m_+_ and m_−_ stand for the active weight loadings of the positive and negative electrodes (g), C_+_ and C_−_ indicate the specific capacitance parameters of those respective electrodes (F cm^−2^), and ΔV_+_ and ΔV_−_ denote the maximum voltage windows for both materials over a full charge–discharge cycle (V), respectively.

For this constructed ASC device, the CV, GCD, and cyclic stability were evaluated within a dual-electrode configuration. Furthermore, the area-normalized capacitance (C_a_, F cm^−2^), energy density (E, mWh cm^−2^), and power density (P, mW cm^−2^) for the assembled device are derived from Equations (1), (3), and (4):

(3) $$E=\frac{C_{a}\times ∆V^{2}}{2\times 3.6}$$

(4) $$P=\frac{3600\times E}{t}$$

where I represent the applied current magnitude (A), Δt refers to the discharge duration (s), S denotes the active electrode area (1 cm^2^), and ΔV defines the working potential range for the ASC.

## 3. Results

### 3.1. Analysis of Structure and Morphology

**Figure 2:**
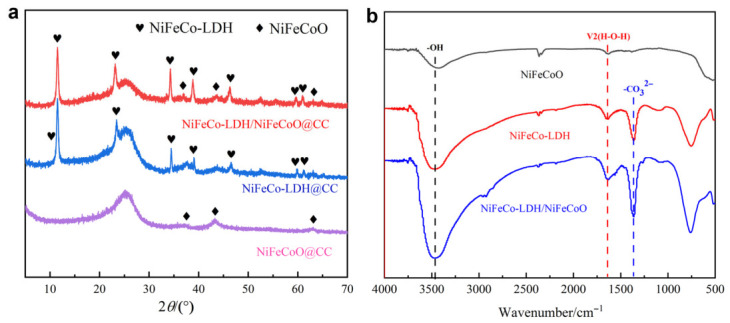
(**a**) XRD patterns of the NiFeCoO@CC, NiFeCo-LDH@CC and NiFeCo-LDH/NiFeCoO@CC samples; (**b**) FT-IR spectra of the NiFeCoO@CC, NiFeCo-LDH@CC and NiFeCo-LDH/NiFeCoO@CC.

Figure 2a shows a diffraction peak at 25.4° corresponding to carbon cloth. According to JCPDS No. 47-1049, the diffraction peaks at 36.93°, 43.71°, and 63.07° can be assigned to the (111), (200), and (220) crystal planes of pure NiFeCoO, respectively [19]. In addition, the peaks located at 11.50°, 23.15°, 34.29°, 38.84°, 46.28°, 59.61°, and 60.94° correspond to the (003), (006), (012), (015), (018), (110), and (113) crystal planes of the NiFeCo-LDH structure, which is consistent with previous report [20]. Notably, all the characteristic peaks corresponding to carbon cloth, NiFeCoO, and NiFeCo-LDH are simultaneously observed in the NiFeCo-LDH/NiFeCoO@CC composite electrode, directly verifying the effective preparation of NiFeCo-LDH/NiFeCoO on carbon cloth.

The functional group compositions of various electrode materials were investigated by FT-IR spectroscopy, and the corresponding spectra are displayed in Figure 2b. The broad and intense absorption peak centered at approximately 3450 cm^−1^ is attributed to −OH stretching vibrations, while the signal at around 1645 cm^−1^ corresponds to the bending mode of surface-adsorbed H_2_O molecules. The characteristic absorption peak at 1360 cm^−1^ originates from CO_3_^2−^ anions, and the bands below 1000 cm^−1^ arise from metal–oxygen (M–O) vibrations. Notably, these −OH groups originate from the host layers of NiFeCo-LDH, whereas the adsorbed water and CO_3_^2−^ are intercalated within the interlayer space. For the NiFeCo-LDH nanosheets, the coexistence of −OH and CO_3_^2−^ confirms successful Fe incorporation into the NiCo-LDH structure. After calcination in air, the CO_3_^2−^ band disappears and the −OH absorption diminishes, indicative of the decomposition of the layered LDH structure and the transformation into NiFeCoO nanosheets. However, upon hydrothermal treatment, the CO_3_^2−^ band reemerges in the NiFeCo-LDH/NiFeCoO composite with a concomitant enhancement of the −OH peak, further confirming the construction of the heterostructure.

**Figure 3:**
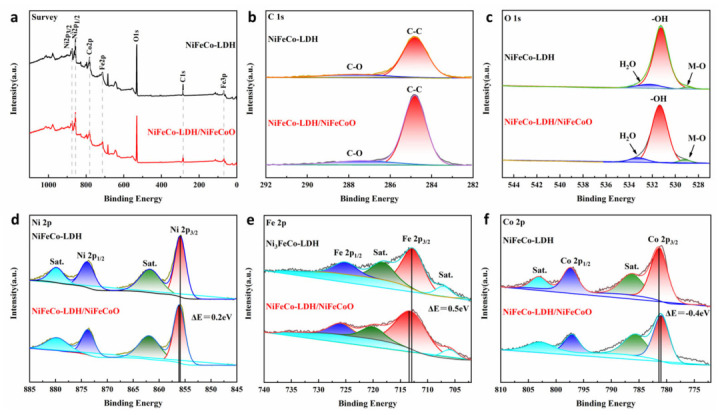
XPS data and corresponding fitting data for Survey (**a**), C 1 s (**b**), O 1 s (**c**), Ni 2p (**d**), Fe 2p (**e**), and Co 2p (**f**) of NiFeCo-LDH@CC and NiFeCo-LDH/NiFeCoO@CC.

The surface composition and chemical states of the specimens were analyzed by XPS, as shown in Figure 3. The signals of Ni, Fe, Co, O, and C elements can be observed from the survey spectra. In the C 1 s spectrum of Figure 3b, the fitted peak at a binding energy of 289.2 eV belongs to the C=O bond. Metal-oxygen bonding, −OH groups, and interlayer adsorbed water in LDH can be observed in the O 1 s spectrum.

The Ni 2p_3_/_2_ peak shifts from 856.0 eV to 856.2 eV after the formation of the heterostructure, indicating an increased valence state of Ni [21]. The Fe 2p_3_/_2_ peak of NiFeCo-LDH/NiFeCoO@CC is located at 713.4 eV, which also shifts to a higher value relative to the pristine NiFeCo-LDH@CC sample (713.0 eV), suggesting an increased valence state of Fe [22,23]. As illustrated by the detailed XPS scan in Figure 3f, the Co 2p_3_/_2_ peak with lower binding energy for NiFeCo-LDH/NiFeCoO@CC appears at 781.0 eV. Since Co^3+^ typically exhibits a lower binding energy relative to Co^2+^ in such configurations, this observation signifies an elevated oxidation level for cobalt, thereby contributing to enhanced electrochemical reactivity [24]. Consequently, these findings demonstrate that the average chemical oxidation states of Ni, Fe, and Co within the composite prove to be elevated relative to the values observed for the pristine NiFeCo-LDH@CC after the formation of the heterostructure, which is favorable for redox reactions and beneficial to supercapacitor performance.

**Figure 4:**
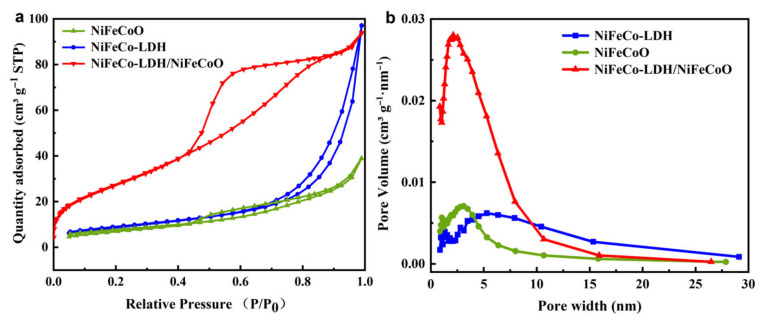
(**a**) Nitrogen physical sorption isotherms alongside (**b**) the derived pore diameter distributions acquired for the as-prepared NiFeCo-LDH/NiFeCoO@CC, NiFeCo-LDH@CC, and NiFeCoO@CC composites.

Figure 4a indicates that the specific surface area of NiFeCo-LDH/NiFeCoO@CC reaches 83.17 m^2^ g^−1^, which is significantly higher than those of NiFeCo-LDH@CC (32.41 m^2^ g^−1^) and NiFeCoO@CC (27.43 m^2^ g^−1^). This result indirectly demonstrates that the introduction of Fe and the construction of the heterojunction can effectively expand the available area of the electrodes [25]. Figure 4b displays the pore size profiles for NiFeCo-LDH/NiFeCoO@CC, NiFeCo-LDH@CC, and NiFeCoO@CC in the mesoporous range (2–30 nm), with average pore sizes of 11.6, 8.4, and 3.6 nm, respectively. The large specific surface area is closely correlated with the charge-storage capability, since it enlarges the contact interface between the electrode and electrolyte, promotes electrolyte infiltration, and thus boosts the electrochemical capacitance.

**Figure 5:**
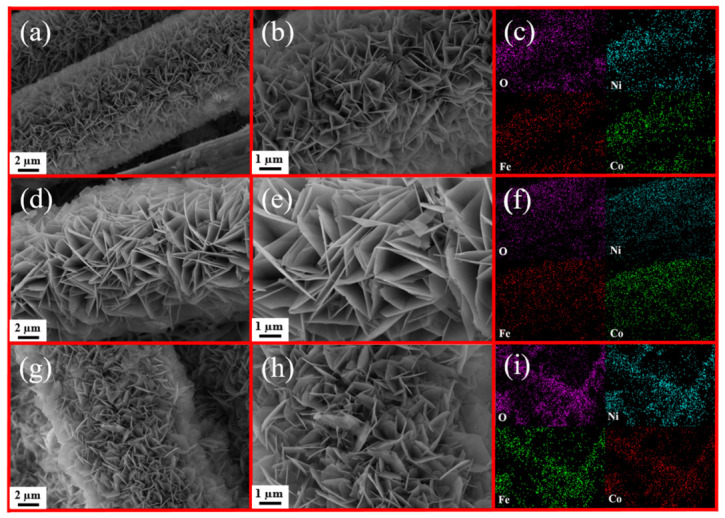
SEM micrographs and EDS elemental maps of (**a**–**c**) NiFeCo-LDH@CC; (**d**–**f**) NiFeCoO@CC and (**g**–**i**) NiFeCo-LDH/NiFeCoO@CC.

The surface morphologies of the prepared samples were examined using SEM. Figure 5a,b shows that numerous interconnected NiFeCoO nanosheets stand vertically on the carbon cloth, providing good mechanical st1ability. EDS mapping further reveals that O, Fe, Ni, and Co are homogeneously distributed on the NiFeCoO surface, as shown in Figure 5c. In Figure 5d,e, NiFeCo-LDH nanosheets are uniformly anchored on the carbon cloth, whose porous structure effectively suppresses the agglomeration of NiFeCo-LDH [26,27]. Subsequently, after further hydrothermal synthesis of NiFeCo-LDH on NiFeCoO@CC, abundant NiFeCo-LDH nanosheets densely cover the original NiFeCoO@CC, forming the novel NiFeCo-LDH/NiFeCoO@CC composite, as shown in Figure 5g,h. The synergistic enhancement of active sites is attributed to internal electric fields, which promote rapid electron transfer. Simultaneously, the interlaced architecture formed by NiFeCoO and NiFeCo-LDH nanosheets facilitates electrolyte infiltration throughout the entire composite [28,29]. Additionally, the EDS data in Figure 5f,i verify an even, unmixed dispersion for O, Fe, Ni, and Co in both the pristine NiFeCo-LDH@CC and the resulting hetero-structured material.

**Figure 6:**
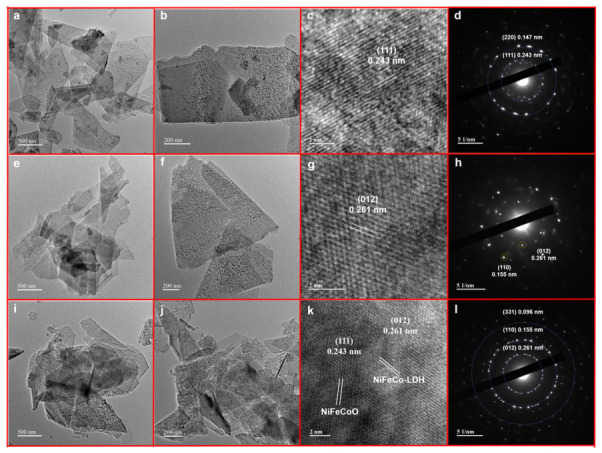
TEM, HRTEM and SAED images of different fabricated samples (**a**–**d**) NiFeCo-LDH; (**e**–**h**) NiFeCoO and (**i**–**l**) NiFeCo-LDH/NiFeCoO.

To further investigate the microstructure, NiFeCoO, NiFeCo-LDH, and NiFeCo-LDH/NiFeCoO were analyzed by TEM. All three samples exhibit a disordered stacked nanosheet morphology. High-resolution TEM reveals a lattice space of 0.243 nm for NiFeCoO, which matches that of the (111) facet (Figure 6c). The distances of the two diffraction rings from the centroid are 0.243 nm and 0.147 nm (Figure 6d), indexing to the (111) and (220) facets. HRTEM characterization of the NiFeCo-LDH sample displays a lattice distance calculated to be 0.261 nm, assigned to the (012) planes (Figure 6g). The distances of the two diffraction rings from the centroid are 0.261 nm and 0.155 nm in Figure 6h, which align with the (012) and (110) planes. Meanwhile, Figure 6i clearly displays disordered stacking of NiFeCoO nanosheets with NiFeCo-LDH. And the combination of HRTEM and SAED can prove the successful establishment of NiFeCo-LDH/NiFeCoO composite structures.

### 3.2. Analysis of Electrochemical Measurements

**Figure 7:**
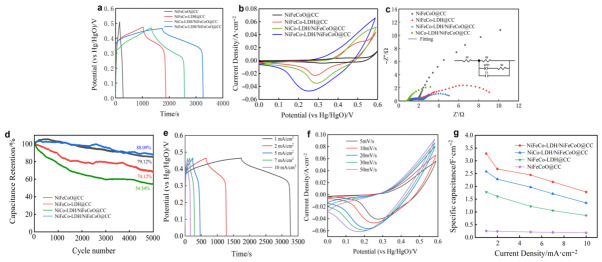
The electrochemical performances of the as-fabricated materials: (**a**) GCD curves of different samples at 2 mA cm^−2^; (**b**) CV curves of different samples at 10 mV s^−1^; (**c**) Nyquist plots; (**d**) Cycling stability of different samples at10 mA cm^−2^; (**e**) GCD curves of NiFeCo-LDH/NiFeCoO@CC at different current densities; (**f**) CV curves of NiFeCo-LDH/NiFeCoO@CC at various scanning rates; (**g**) Specific capacitances of different samples at different current densities.

Firstly, the GCD curves in Figure 7a show the electrochemical performance of the different electrodes. Combining the GCD curve with Equation (1), NiFeCo-LDH/NiFeCoO@CC delivers the highest areal specific capacitance of 3.282 F cm^−2^, better than NiCo-LDH/NiFeCoO@CC (2.582 F cm^−2^), NiFeCo-LDH@CC (1.777 F cm^−2^) and NiFeCoO@CC (0.254 F cm^−2^). Table 1 [30,31,32,33,34] shows that the NiFeCo-LDH/NiFeCoO@CC exhibits a clear advantage in area specific capacitance. The improved capacitive performance of NiFeCo-LDH/NiFeCoO@CC mainly stems from the higher oxidation states of Ni, Co, and Fe within the LDH framework, which boost the redox activity. Additionally, the heterostructure constructed by NiFeCo-LDH and NiFeCoO can also accelerate the electron and ion transport kinetics during the redox reactions.

The four CV curves display distinct redox peaks, which are attributed to the reversible Faradaic reactions corresponding to M(OH)_2_/MOOH (where M denotes Ni, Co, and Fe) [35,36]. Notably, the NiFeCo-LDH/NiFeCoO@CC composite exhibits the largest CV integral area and the strongest redox peak intensity, indicating its higher specific capacitance compared with the other tested electrodes [37]. This performance enhancement is mainly attributed to the rapid electron transfer at the heterojunction interface and the synergistic effect of multiple Faradaic redox reactions within the ternary LDH. Furthermore, the relevant reversible reactions are as follows [38]:

Co(OH)_2_ + OH^−^ ⇌ CoOOH + H_2_O + e^−^ (5)

CoOOH + OH^−^ ⇌ CoO_2_ + H_2_O +e^−^ (6)

Ni(OH)_2_ + OH^−^ ⇌ NiOOH + H_2_O + e^−^ (7)

Fe(OH)_2_ + OH^−^ ⇌ FeOOH + H_2_O + e^−^ (8)

According to the Nyquist plots (Figure 7c) and the data in Table 2, the NiFeCo-LDH/NiFeCoO@CC composite displays the lowest equivalent series resistance (Rs), which indicates an accelerated interfacial electron conduction promoted by the heterojunction. Additionally, this sample presents the smallest high frequency semicircular arc among all evaluated materials, reflecting the interfacial charge-transfer resistance (Rct) across its solid and liquid boundary [39]. NiFeCo-LDH/NiFeCoO@CC exhibits the lowest Rct, indicating higher conductivity and charge-transfer capability, which is consistent with the CV analysis. Thus, the multistructure of the composites facilitates electron transport between active materials and the electrolyte, implying enhanced charge storage.

Continuous GCD measurements were executed at 10 mA cm^−2^ across 5000 cycles to assess the extended cycling endurance, which serves as an essential metric for real-world implementation (Figure 7d). Among these, NiFeCo-LDH/NiFeCoO@CC maintained a capacitance retention of 88.09% after 5000 cycles. This can be attributed to the synergistic composite structure of NiFeCo-LDH and NiFeCoO, which effectively enhances structural stability and suppresses the degradation and structural collapse of LDH nanosheets during continuous charge–discharge cycling [40]. It should be noted that the slight upward trend in capacity during the early stages is primarily driven by the progressive electrochemical activation of the inner active sites [41], while the subsequent decay in specific capacity may result from the detachment of active materials from the carbon cloth during prolonged cycling. Figure 7e presents the GCD curves of NiFeCo-LDH/NiFeCoO@CC at various current densities. When the current density decreased from 10 mA cm^−2^ to 1 mA cm^−2^, the areal capacitance increased from 1.779 F cm^−2^ to 3.282 F cm^−2^, demonstrating excellent rate capability. As illustrated in Figure 7f,g, all CV curves maintained similar shapes when the scan rate was varied from 2 mV s^−1^ to 50 mV s^−1^, indicating outstanding reversibility [42].

**Figure 8:**
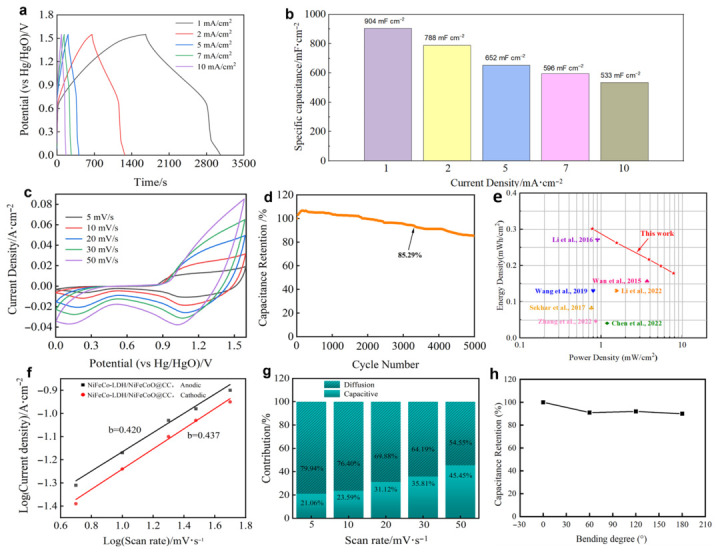
Electrochemical performance of NiFeCo-LDH/NiFeCoO@CC//AC ASC device: (**a**) GCD curves at various current densities; (**b**) comparison of the areal capacitance at various current density; (**c**) CV curves at various scan rate; (**d**) Cycling stability at 10 mA cm^−2^; (**e**) Ragone plots of the ASC; (**f**) Determination of b-value from the cathodic and anodic peak currents of the ASC; (**g**) Relative contribution of the diffusion and capacitive-controlled charge storage at different scan rates of the ASC; (**h**) Capacitance retention at various bending degree [30,31,32,33,34,43,44].

Practical energy storage performance of the NiFeCo-LDH/NiFeCoO@CC electrode was evaluated by assembling a full ASC. As shown in Figure 8a, the symmetric voltage plateaus in the GCD curves demonstrate favorable electrochemical reversibility and an efficient Faradaic redox process. Moreover, the areal capacitance decreases from 0.904 F cm^−2^ to 0.535 F cm^−2^ with the current density increasing from 1 mA cm^−2^ to 10 mA cm^−2^ in Figure 8b, reflecting satisfactory rate capability.

According to Figure 8c, the CV curves for this assembled ASC maintain a consistent polygonal geometry across various sweep speeds, further verifying the outstanding reversibility of the device. Benefiting from the robust heterostructure and the inherent stability of NiFeCoO, the full cell successfully retains 85.29% of its initial capacity following 5000 continuous cycles, showcasing exceptional long-term durability in Figure 8d. Beyond durability, the energy and power outputs serve as crucial performance metrics for the assembled device. The NiFeCo-LDH/NiFeCoO@CC//AC device presents an excellent power density of 0.776 mW cm^−2^ at an energy density of 0.302 mWh cm^−2^, and maintains 0.179 mWh cm^−2^ at a maximum power density of 7.773 mW cm^−2^, implying great applied potential in mobile electronic devices. Furthermore, the Ragone plot [30,31,32,33,34,43,44] displays the variation in energy density as a function of power density in Figure 8e.

Based on the CV curves recorded at various scan rates in Figure 8f, the kinetics of the charge-storage behavior is analyzed using the cathodic and anodic peak currents (i) and scan rate (v), according to Equation (9) [45]:

(9) $$i=av^{b}$$

where a and b are fitted variable. b = 1 implies capacitive charge storage, while b = 0.5 indicates the intercalation mechanism. In Figure 8f, the b values of the anodic and cathodic peaks are calculated to be 0.42 and 0.437, respectively, which reveals typical battery-type behavior. Accordingly, the quantitative contributions from pseudocapacitive and diffusion-controlled processes can be analyzed on the basis of Equation (10) [46]:

(10) $$i\left(V\right)=k_{1}v+k_{2}v^{1/2}$$

According to the formula, the contribution of NiFeCo-LDH/NiFeCoO@CC//AC capacitance control and diffusion control is calculated in Figure 8g. As the scan rate increases from 5 to 50 mV s^−1^, the capacitive contribution of the NiFeCo-LDH/NiFeCoO@CC//AC electrode increases from 21.27% to 45.45%, indicating efficient charge storage capacity. Moreover, the structural stability of the device was assessed through electrochemical tests under different bending states, with corresponding results displayed in Figure 8h. Notably, the areal capacitance does not change significantly during 180° bending. These results confirm that the as-fabricated asymmetric device integrates superior energy-storage performance and favorable mechanical flexibility, showing high promise for practical applications.

**Figure 9:**
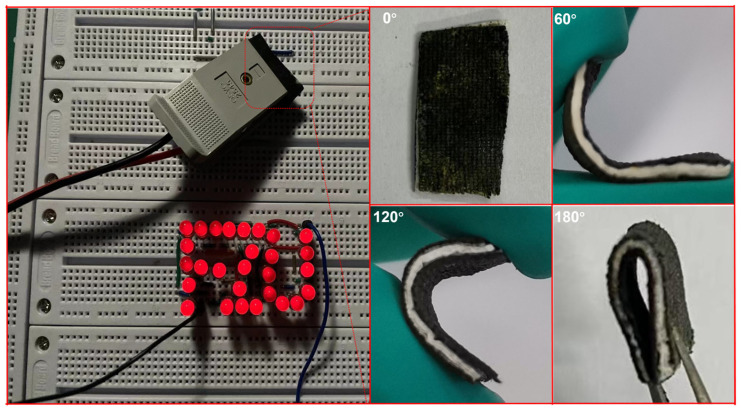
The “FZU” logo composed of red LEDs driven by the integrated ASC system.

To verify the practical application potential of the NiFeCo-LDH/NiFeCoO@CC electrode, the as-fabricated NiFeCo-LDH/NiFeCoO@CC//AC asymmetric supercapacitor successfully lights up LEDs in an “FZU” pattern, as shown in Figure 9. Meanwhile, the NiFeCo-LDH/NiFeCoO@CC//AC@CC electrode was bent at various angles (0°, 60°, 120°, and 180°) and can rapidly restore its original state after folding, while retaining the ability to light up LEDs. These observations confirm the exceptional mechanical robustness and promising real-world applicability of the as-fabricated solid-state device.

**Figure 10:**
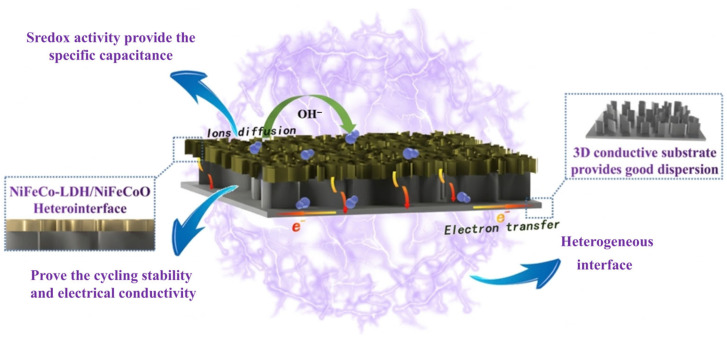
Synergistic advantages of the hierarchical NiFeCo-LDH/NiFeCoO@CC electrode for supercapacitors.

Considering the above experimental results and analysis, the NiFeCo-LDH/NiFeCoO@CC composite exhibits remarkable performance as an anode material for supercapacitors, mainly attributed to the following aspects. Firstly, NiFeCoO nanosheets improve the cycling stability and electrical conductivity of the composite, while also providing part of the specific capacitance. Secondly, NiFeCo-LDH nanosheets with strong redox activity provide the main specific capacitance. The constructed heterogeneous interface effectively accelerates electron and ion transport during the entire electrochemical process. Furthermore, the supporting effect of NiFeCoO nanosheets alleviates the structural collapse of NiFeCo-LDH nanosheets, thus enhancing the cycling stability of the composite [47]. These synergistic effects contribute to the improved capacitance and stability of the NiFeCo-LDH/NiFeCoO@CC electrode. Figure 10 illustrates the structural merits of this hierarchical architecture.

## 4. Conclusions

To comprehensively promote the electrochemical performance of NiCo-LDH in supercapacitor applications, a novel NiFeCo-LDH/NiFeCoO@CC composite featuring a multi-structured architecture was successfully synthesized via a facile hydrothermal method. The results indicate that each component in the composite plays a key role in enhancing electrical conductivity, facilitating electron transfer, raising charge storage capability, and strengthening cycling durability. In particular, the heterostructure constructed between NiFeCo-LDH and NiFeCoO effectively strengthens the synergistic effect inside the composite. Accordingly, the NiFeCo-LDH/NiFeCoO@CC electrode achieves a remarkable areal capacitance of 3.282 F cm^−2^ at 1 mA cm^−2^, which is nearly three times higher than that of conventional NiCo-LDH, together with a superior capacitance retention of 88.09% after 5000 cycles at 10 mA cm^−2^. Moreover, the as-assembled NiFeCo-LDH/NiFeCoO@CC//AC asymmetric supercapacitor realizes a high energy density of 0.302 mWh cm^−2^ at a power density of 0.776 mW cm^−2^ and retains 85.29% of its initial capacitance after 5000 consecutive charge–discharge cycles at 10 mA cm^−2^. Benefiting from its unique multi-structured architecture and the increased valence states of the metal elements, the NiFeCo-LDH/NiFeCoO@CC composite shows outstanding potential as a high-performance electrode material for supercapacitors. This work presents a simple and effective strategy for the rational design of multi-structured and high-performance energy storage materials.

## Figures and Tables

**Table 1:** Performance of NiFeCo-LDH/NiFeCoO@CC compared with reported studies.

Materials	Rate (mA cm^−2^)	C_a_ (F cm^−2^)	Ref.
NC LDH NSs@Ag@CC	1	1.133	[30]
Co_9_S_8_@PPy@NiCo-LDH NTAs@CC	1	2.650	[31]
NiCo_2_S_4_@NiMn-LDH/GS	1	1.740	[32]
CC@ZnO@NiCo-LDH	1	0.667	[33]
PBA@Ni_0.4_Co_0.6_-LDH/NF	1	2.004	[34]
NiFeCo-LDH/NiFeCoO@CC	1	3.282	This work

**Table 2:** Rs and Rct parameters for the tested materials.

Sample	Rs Series Resistance (Ω)	Rct Charge Transfer Resistance (Ω)
NiFeCoO@CC	0.602	5.877
NiFeCo-LDH@CC	0.428	1.832
NiCo-LDH/NiFeCoO@CC	0.359	1.2136
NiFeCo-LDH/NiFeCoO@CC	0.306	0.7042

## Data Availability

The original contributions presented in this study are included in the article. Further inquiries can be directed to the corresponding author.
